# Modeling Noise-Related Timbre Semantic Categories of Orchestral Instrument Sounds With Audio Features, Pitch Register, and Instrument Family

**DOI:** 10.3389/fpsyg.2022.796422

**Published:** 2022-04-01

**Authors:** Lindsey Reymore, Emmanuelle Beauvais-Lacasse, Bennett K. Smith, Stephen McAdams

**Affiliations:** Department of Music Research, Schulich School of Music, McGill University, Montreal, QC, Canada

**Keywords:** timbre, audio features, timbre and noise perception, timbre analysis, register, extended technique, musical instrument timbre, timbre semantics

## Abstract

Audio features such as inharmonicity, noisiness, and spectral roll-off have been identified as correlates of “noisy” sounds. However, such features are likely involved in the experience of multiple semantic timbre categories of varied meaning and valence. This paper examines the relationships of stimulus properties and audio features with the semantic timbre categories *raspy/grainy/rough*, *harsh/noisy*, and *airy/breathy*. Participants (*n* = 153) rated a random subset of 52 stimuli from a set of 156 approximately 2-s orchestral instrument sounds representing varied instrument families (woodwinds, brass, strings, percussion), registers (octaves 2 through 6, where middle C is in octave 4), and both traditional and extended playing techniques (e.g., flutter-tonguing, bowing at the bridge). Stimuli were rated on the three semantic categories of interest, as well as on perceived playing exertion and emotional valence. Correlational analyses demonstrated a strong negative relationship between positive valence and perceived physical exertion. Exploratory linear mixed models revealed significant effects of extended technique and pitch register on valence, the perception of physical exertion, *raspy/grainy/rough*, and *harsh/noisy*. Instrument family was significantly related to ratings of *airy/breathy*. With an updated version of the Timbre Toolbox (R-2021 A), we used 44 summary audio features, extracted from the stimuli using spectral and harmonic representations, as input for various models built to predict mean semantic ratings for each sound on the three semantic categories, on perceived exertion, and on valence. Random Forest models predicting semantic ratings from audio features outperformed Partial Least-Squares Regression models, consistent with previous results suggesting that non-linear methods are advantageous in timbre semantic predictions using audio features. Relative Variable Importance measures from the models among the three semantic categories demonstrate that although these related semantic categories are associated in part with overlapping features, they can be differentiated through individual patterns of audio feature relationships.

## Introduction

Several audio features have been identified as correlates of “noisy” sounds, including inharmonicity and measures of spectral shape such as spectral flatness, spectral centroid, and spectral roll-off (McAdams et al., 2004; Alluri and Toiviainen, 2010; Wallmark, 2014; Wallmark et al., 2018; Caetano et al., 2019). However, not all types of noise are semantically equal: when timbre categories are nuanced, features associated with “noisy” sounds may be correlates of multiple semantic categories with varied meanings and even varied valence. Reymore and Huron (2020) built a 20-dimensional model of musical instrument timbre qualia from the results of interviews and rating tasks. This model included three timbre dimensions plausibly associated with noise-related audio features—*shrill/harsh/noisy*, *raspy/grainy* and *airy/breathy*—whereas a further two dimensions appeared to potentially refer to harmonicity and/or a lack of “noisy” features—*pure/clear* and *focused/compact*. Speculating on correlates of these semantic categories, Reymore and Huron (2020) noted that although noise has often been associated with negative valence and high physical exertion (as in Wallmark et al., 2018), noise components in breathy timbres, typically measured in speech research with harmonic-to-noise ratio (HNR), may convey a sense of proximity or intimacy that carries positive valence. HNR is an assessment of the ratio between periodic and non-periodic components comprising an acoustic signal (Keating et al., 2015). Thus, a feature such as HNR may be relevant to multiple semantic categories. In general, although audio features may correlate with multiple semantic categories, we propose that certain combinations of audio features may create distinctive, perceptible patterns for listeners that are associated with varying, nuanced semantic categories.

Among the available features, several have been associated with noise in previous literature and so were of particular interest for the interpretation of our results, including inharmonicity, noisiness, noise energy, spectral flatness, and HNR. Analogous phonetic processes of the voice, such as breathy and creaky voice, may also offer insight into the audio feature correlates. Work by Keating et al. (2015) found that different audio features or combinations of these features characterized different varieties of creaky voice. The researchers examined HNR as part of a set of features used to characterize different varieties of creaky voice. Features that characterized prototypical creaky voice in Keating et al. (2015) include a lower and more irregular F0 (i.e., jitter), low HNR, and a small difference between the amplitudes of the first and second harmonics (H1–H2). The presence of any of these features was enough for a voice to sound creaky. Similar research has been conducted for breathy voice. The best predictors of breathiness ratings in Hillenbrand et al. (1994) were the amplitude of the first harmonic, periodicity in band-limited signals, and aspiration noise.

Wallmark (2014), Wallmark et al. (2018) provides an ethological account of noisy timbre, positing that the overdrive of voices (screaming, wailing, growling, etc.) tends to happen in response to extremely negative stimuli that provoke high arousal. There are ethological reasons to suppose that the processing of noisy timbres may recruit phylogenetically ancient pathways in addition to offering an adaptive advantage. With the MIR Toolbox, Wallmark et al. (2018) assessed spectral and temporal features including high frequency energy, spectral centroid, inharmonicity, spectral flatness, zero-crossing rate, and auditory roughness. They found that each spectral parameter exhibited a strong correlation with at least one of the perceptual measures, such as noisiness, anger, and exertion. This research also found that activity in somatomotor areas of the brain increased with negatively valenced (disliked) sounds.

Human screams are correlated with high pitch, roughness, wide fundamental frequency range, and narrow bandwidth, but the role of roughness in classifying screams seems to be dependent in part on pitch (Schwartz et al., 2019). Trevor et al. (2020) found that the music in horror films mimics the acoustic features (roughness) of human screams. Both screams and scream-like music exhibited a higher level of roughness, had higher arousal, and had lower valence ratings than non-scream-like music and vocalizations. These results provide converging evidence for Wallmark’s embodied account of timbre, which argues that “noisy” timbre perception is fundamentally motor mimetic.

Physical exertion leads to timbral abnormalities often heard as noisy, leaving acoustic traces of high-frequency energy, inharmonicity, flat spectrum, and roughness. Despite this, Wallmark (2014) notes that timbral noise does not have a single, consistently agreed-upon correlate, though inharmonicity and spectral flatness are related to the perception of noise. Noisy timbre may be perceived as an index for heightened bodily arousal and exertion. This, in turn, may influence how positively or negatively a sound is rated. Following Wallmark et al. (2018), the current study tests whether there exists a correlation between perceived physical exertion and valence in semantically “noisy” timbres. Specifically, we hypothesize that valence ratings will be negatively correlated with ratings for the semantic category *harsh/noisy* and that these sounds are more likely to be perceived as having been produced with more physical exertion (blowing, plucking, or bowing with more effort than what would be considered normal for the instrument). Note that in adapting this semantic category from the Reymore and Huron (2020) model, because the stimuli in the current study vary in pitch register, we removed the word “shrill” due to its association with high pitch.

Wallmark (2014) focused on the extremes of vocal production in his discussion of noisy timbres, but “noise” in timbre may not only be produced at the extremes. Reymore and Huron’s model suggests that audio features related to noise may be very important in deriving meaning from timbres in a range of semantic categories. Some of the same audio features or combinations thereof that are associated with the traditional, negatively valenced concept of noise may also be associated with other nuanced semantic categories, some of which may be positively or neutrally valenced and carry different meanings.

For example, the *raspy/grainy/rough* timbral category may not necessarily be associated with extreme physical exertion. In a voice, raspiness could signify age, whether someone smokes cigarettes, vocal fatigue, or even sultriness. Such terms also describe the phenomenon of vocal fry, or creaky voice, which has become a recent topic of research, particularly in relation to the use of this vocal register by young, American women (Dallaston and Docherty, 2020). Yuasa (2010) found that two populations of college-aged Americans perceived vocal fry as “hesitant, non-aggressive, and informal, but also educated, urban-oriented, and upwardly mobile” (p. 315). In contrast, Anderson et al. (2014) found that among adult Americans, vocal fry was perceived as “less competent, less educated, less trustworthy, less attractive, and less hirable” in comparison to a normal speaking voice. Given that some associations with raspy or creaky voice are unpleasant or negative, but also that associations may be context dependent, we did not pose a directional prediction for valence ratings of *raspy/grainy/rough* sounds.

The *harsh/noisy* semantic timbre category may be more intuitively associated with negative valence and physical exertion. *Harsh* may be associated with emotional negativity, which may influence valence ratings of *harsh/noisy* sounds. For example, Morton (1977), in his article on sounds produced by birds and mammals, uses the terms “harsh” to describe sounds associated with hostile motivation (e.g., aggression). Wallmark et al. (2018) found that ratings of “noisiness” were negatively correlated with valence and positively correlated with perceived exertion.

People naturally modify the breathiness of the voice relative to the physical distance to the person they are talking to, and thus breathy voice quality contributes to a sense of acoustic intimacy, conveying proximity and/or coziness (Huron, 2013). The whispered voice presents the most extreme example of this, and the timbre of the whisper has been linked to the experience of intimacy in studies of the autonomous sensory meridian response (ASMR; Andersen, 2015). Kovacevich and Huron (2018) present evidence of similarities between physiological responses to ASMR and musically induced frisson or chills, both of which are typically considered highly pleasurable by those who experience them. Warrenburg et al. (2021) propose a theory of sonic intimacy in music, linking close-miking and sounds of whispering/breathing with feelings of intimacy and closeness, which may work to mitigate experiences of social isolation. We predicted that *airy/breathy* sounds are associated with less exertion and that ratings for *airy/breathy* would be positively correlated with valence ratings, perceptions that may be linked to the concept of physical intimacy or closeness.

In sum, this experiment identifies audio feature correlates for various types of noise in musical instrument timbres and distinguishes the connotations associated with these semantic noise categories in relation to valence and exertion. The current experiment examined three modified semantic categories derived from Reymore and Huron’s model: *raspy/grainy/rough*, *harsh/noisy*, and *airy/breathy*. Our aims were to determine whether these categories share audio correlates and how these categories can be distinguished based on their underlying audio features. We first collected semantic ratings on a set of sounds produced by orchestral instruments. Next, we used linear and non-linear approaches to model the mean semantic ratings using audio features, with the goal of uncovering distinctive audio signatures for each semantic category. For modeling, we used spectral and harmonic features from a recently updated version of the Timbre Toolbox (R-2021A; Kazazis et al., 2021).

## Materials and Methods

### Participants

Participants (*n* = 161; *F* = 95, *M* = 57, other = 1), were recruited using the Internet platform Prolific. Data from eight participants were excluded due to one or more of the following reasons: admission that headphones were not used or not working properly during the experiment, timing indicating that instructions were not read, or evidence of random responses, such as repetition of the same response over a long sequence of successive trials or ratings that were poorly correlated with the rest of the participants. In total, data from 153 participants were included in the final analysis, representing 51 complete sets of responses to the stimulus set. As identified through Prolific’s screening process, all participants were native English speakers. Participants were on average 32 years of age (*SD* = 11.2, range = 18–68); 41 of the 153 self-identified as musicians using the single-question measure from the Ollen Musical Sophistication Index (Ollen, 2006). All subjects gave written informed consent in accordance with the Declaration of Helsinki. The protocol was certified for ethics compliance by the McGill University Research Ethics Board II. Participants were compensated for their participation.

### Stimuli

The stimulus set consisted of 156 approximately 2-s sound clips of single notes (pitch class C) played by various orchestral instruments, normalized and matched for loudness by the researchers. Sounds were taken from three sound banks: Vienna Symphonic Library (VSL, Vienna Symphonic Library GmbH, 2011), McGill University Master Samples (MUMS, Opolko and Wapnick, 1987) and the conTimbre library (Hummel, 2014). Because our goal was exploratory modeling of semantic categories, we aimed to sample stimuli as widely as possible from the semantic space of interest—that is, to sample sounds representing high, moderate, and low ratings on all categories of interest. We began by listening to many sounds from the three sample libraries and selecting examples we felt represented the widest possible range of likely ratings on *airy/breathy*, *harsh/noisy*, and *raspy/grainy/rough*. The stimulus selection process was then further guided by the results of a pilot study (*n* = 10) of 46 sounds. The final stimulus set included 42 instruments playing in five registers (C2–C6, where C4 is middle C with a fundamental frequency of 262 Hz) using both traditional and extended playing techniques. Examples of extended techniques represented include flutter-tonguing, growls, and bowing at the bridge. More details about the stimuli are included in the Supplementary Material.

### Procedure

To avoid an overly long experiment, each of the 153 participants rated one-third of the stimulus set (52/156 sounds), resulting in 51 complete sets of rating data on the entire stimulus set. To ensure that all sounds were rated an equal number of times, each complete set was the union of three participants’ ratings. For each group of three participants, the entire stimulus set was randomly separated into three disjoint subsets, one for each participant. Sounds were heard in a random order.

Participants took part in the online experiment using their personal computer. Once a participant opted to take part in the experiment, they were routed from Prolific to a custom online experimental interface implemented in JavaScript and hosted on a web server in the Music Perception and Cognition Lab at McGill University. After providing informed consent, participants were presented with instructions and listened once to all stimuli presented in a random order before beginning the experiment. Instructions stressed that headphones should be used throughout the experiment, and participants were required to confirm that they were using headphones before beginning the study. At the end of the study, participants answered demographic questions, including questions about musical background and were required to report the make and model of headphones used during the study.

During the study, participants rated how applicable each semantic category (*raspy/grainy/rough*, *harsh/noisy*, *airy/breathy*) was to a given stimulus using a continuous sliding scale from 1 (*does not describe at all*) to 7 (*describes extremely well*), where the midpoint was labeled *describes moderately well*. Participants also rated valence (*negative* to *positive*) and perceived playing exertion (*little to no exertion* to *high exertion*). Ratings were made in separate blocks for each scale, so participants rated their stimulus subset a total of five times. At the beginning of each trial (except the first in each block), the stimulus was automatically played, and participants could play it again as many times as desired. The presentation order of the scales and the stimuli within each block were randomized. The experiment took approximately 30 min to complete.

## Results

### Rating Consistency and Correlation Analyses

First, we conducted reliability analyses and correlations among the scales. Interrater reliability was calculated among complete sets of ratings, where each set included ratings from three participants using the *alpha* function in the *psych* package (Revelle, 2020) in R (R Core Team, 2020; version 4.0.5). Overall interrater reliability was strong (Cronbach’s α = 0.93 for *airy/breathy*, 0.95 for *raspy/grainy/rough*, 0.97 for *harsh/noisy*, 0.95 for valence, and 0.87 for exertion). The ratings were then averaged per stimulus prior to conducting further correlational analyses. Table 1 reports correlations among the five scales; all *p*-values reported in this paper use a Holm correction as implemented by the *corr.test* function in the *psych* package (Revelle, 2020).

**TABLE 1:** Pearson’s correlation coefficients among ratings of perceived valence, playing exertion, *airy/breathy*, *raspy/grainy/rough*, and *harsh/noisy*.

	Airy/breathy	Raspy/grainy/ rough	Harsh/noisy	Valence
Raspy, grainy, rough	–0.09			
Harsh, noisy	–0.54***	0.53***		
Valence	0.31***	–0.90***	–0.61***	
Exertion	–0.04	0.50***	0.46***	–0.48***

df = 154, Holm-corrected, ***p < 0.001.

Results supported our hypothesis that valence and exertion would be negatively correlated. We had also predicted that exertion and valence would be associated differently with various semantic terms: (1) ratings of *airy/breathy* would be correlated positively with valence and negatively with exertion, (2) *raspy/grainy/rough* ratings would correlate moderately positively with exertion (with no directional prediction for the correlation with valence), and (3) *harsh/noisy* would be correlated negatively with valence and positively with exertion. *Airy/breathy* was not correlated significantly with exertion as we had predicted, but *raspy/grainy/rough* and *harsh/noisy* were both moderately correlated with exertion. Contrary to our expectations, *raspy/grainy/rough* was more negatively correlated with valence than was *harsh/noisy* {comparison using the *cocor* package with Zou’s (2007) confidence interval; 95% CI [0.20,0.40]}. As predicted, *airy/breathy* was moderately positively correlated with valence.

### Linear Mixed Model Analyses

Exploratory linear mixed modeling was used to create five models predicting mean ratings of valence, exertion, and the three semantic descriptors using register, instrument family, and extended technique as categorical predictor variables. Modeling was carried out with the *lmer* function in the *lme4* package (Bates et al., 2015) in R. Because each stimulus was rated by each participant in a repeated-measures design, a mixed modeling approach was necessary to account for variance due to participants and stimuli. For each of the five models, the random structure was established first by comparing two models containing all three variables of interest and all interactions; the first model included only random intercepts for participant and stimulus, whereas the second model used the maximal random effects structure (random intercepts for participant with random slopes for register, instrument family, and technique and random intercepts for the stimuli). This maximal random effects structure was derived from that of similar linear mixed models described in McAdams et al. (2017; see also Barr et al., 2013). Next, these pairs of models (effects only vs. effects and slopes) were compared using a log likelihood ratio test via the *anova* function. In all five cases, the added random slopes significantly improved model fit, and so we retained the maximal random effects structure in subsequent modeling.

The fixed structure of the models was determined using the *dredge* function from the *MuMIn* package (Barton, 2009), which generates tables of models with combinations of fixed effect terms in a global model. The *dredge* function provides model rankings based on various possible criteria; we chose to examine rankings based on AICc, AIC, and BIC. For each predicted scale, models including main effects of register, instrument family, extended technique, and all interactions, plus random effects and slopes as described above, were run through the *dredge* function, which assessed all possible versions of the models with combinations of the specified fixed effects. For all five models, all three information criteria converged to suggest models that included the three variables of interest (instrument family, register, extended technique) without interaction terms.

**FIGURE 1:**
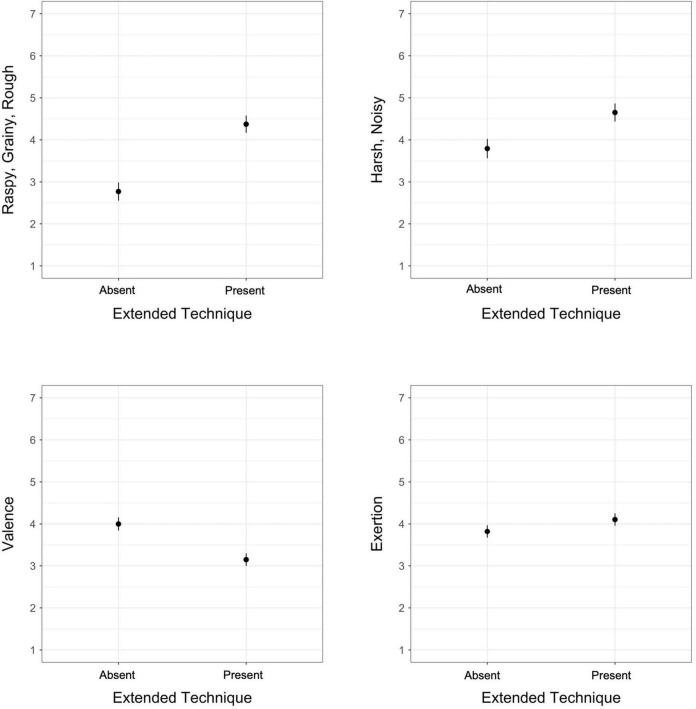
Estimated marginal means for the absence/presence of an extended technique in models of exertion, valence, *raspy/grainy/rough*, and *harsh/noisy*; vertical bars represent 95% confidence intervals.

**FIGURE 2:**
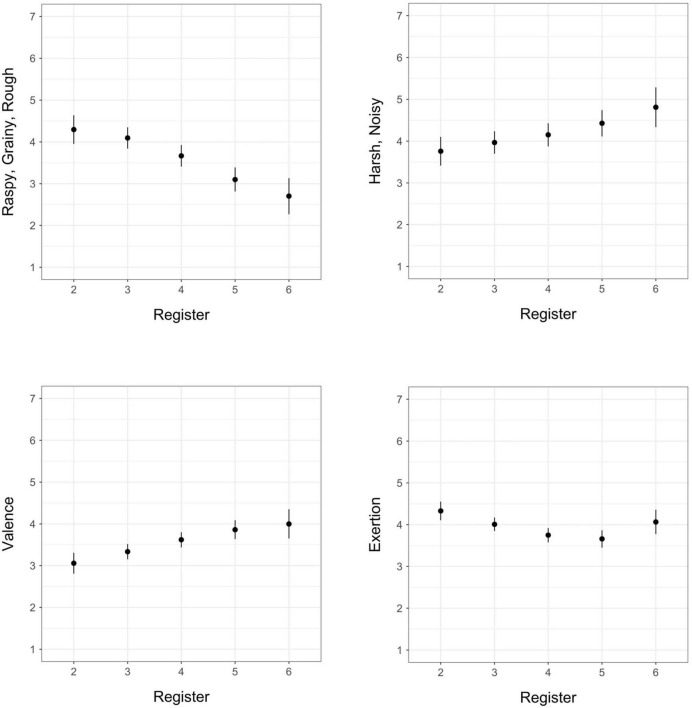
Estimated marginal means for register (octave) in models of exertion, valence, *raspy/grainy/rough*, and *harsh/noisy*; vertical bars represent 95% confidence intervals.

**FIGURE 3:**
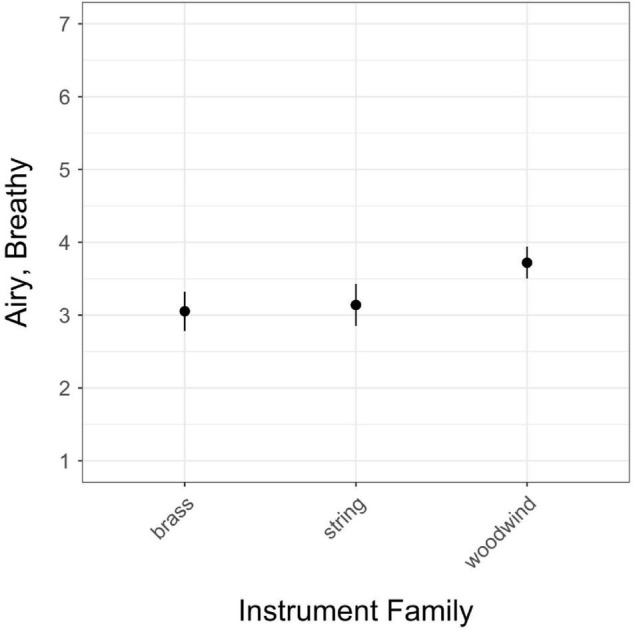
Estimated marginal means for instrument family in the *airy/breathy* model; vertical bars represent 95% confidence intervals.

The resulting models were subjected to Type III Wald χ^2^-tests; these analyses are reported in Table 2 and estimated marginal means are visualized in Figures 1–3. Note that for the purposes of these models, the fixed effect of “extended technique” was operationalized as any non-typical or modified production of a sound by an instrument. It should also be noted that in building the stimulus set, we had deliberately chosen stimuli which used extended techniques that we deemed especially rough, harsh, noisy, etc. for the purpose of sampling sounds from as wide of a semantic space as possible relative to our semantic categories of interest. Thus, although all extended techniques do not necessarily produce “harsh” or “rough” or “airy” sounds, we expected that in the context of this experiment, extended playing techniques would be related to our dependent variables. Percussion sounds were removed from the stimulus set prior to conducting the linear mixed model analysis because of their low number (*n* = 9) relative to woodwind, brass, and string instrument sounds.

**TABLE 2:** Effects of the factors register (R), extended technique (ET), and instrument family (F) on each semantic category, as well as on valence and exertion.

	Airy/breathy		Raspy/grainy/rough		Harsh/noisy	
	*χ^2^*	*p*	*χ^2^*	*p*	*χ^2^*	*p*
Intercept	1257.45	<0.001***	1826.71	<0.001***	2214.71	<0.001***
*R*	3.49	0.48	48.08	<0.001***	14.81	0.005**
ET	0.72	0.40	137.00	<0.001***	37.61	<0.001***
*F*	22.95	<0.001***	1.85	0.39	1.92	0.38

	**Valence**		**Exertion**			
	***χ^2^***	***p***	***χ^2^***	***p***		

Intercept	3421.90	<0.001***	3721.21	<0.001***		
*R*	22.99	<0.001***	27.95	<0.001***		
ET	78.32	<0.001***	13.27	<0.001***		
*F*	4.34	0.11	3.58	0.17		

**p < 0.01; ***p < 0.001.

Sounds produced using extended techniques received significantly higher ratings on *raspy/grainy/rough*, *harsh/noisy*, and perceived exertion, but significantly lower ratings on valence. *Raspy/grainy/rough* demonstrated the largest difference in ratings between the two conditions (Figure 1). These results for exertion reflect the findings of Wallmark et al. (2018): the authors noted that perceived exertion in that experiment was greater for distorted guitar timbres even though the production of these sounds does not actually require the musician to use increased exertion. In parallel, listeners in our study rated sounds created with extended techniques as requiring more exertion than those produced with traditional means, despite the reality that many of the techniques do not require more exertion to achieve (and in some cases may even require less exertion).

Positive valence and *harsh/noisy* increased with increasing register, *raspy/grainy/rough* decreased with increasing register, and exertion ratings demonstrated a concave relationship with register (Figure 2). *Airy/breathy* showed no significant pattern with register. The opposite relationships we observed between *raspy/grainy/rough* and *harsh/noisy* with respect to valence demonstrate that register may be a key factor in differentiating these two timbre qualia.

Our observed increasing linear trend of valence and register represents a different finding from that of McAdams et al. (2017), who observed a non-linear, convex relationship of valence and register (except in the percussion family) with the sixth octave having lower predicted mean valence (preference) ratings across instruments than the fifth. This difference in findings may in part be due to the distribution of extended technique sounds across registers in our stimulus set: overall our set included relatively fewer examples in the 6th register and fewer examples within this register that used extended techniques. It may also be possible that extended techniques in general tended to be less noticeable or produce less unpleasant effects with increasing register.

Although the relationship between register and valence in our stimulus set was linear, we observed a slight non-linear concave pattern in perceived exertion over register, with the lowest predicted mean exertion rating at C5. Considering tension arousal from the (McAdams et al., 2017) experiment as a roughly parallel comparison for exertion ratings in the current study, our observed pattern is consistent with their results, though the overall nadir for tension arousal in their study seems to be lower in register than that of exertion in this study.

Finally, the only significant difference observed among instrument families (woodwind, brass, string) was in ratings of *airy/breathy* (Figure 3), in which woodwinds were rated as higher on *airy/breathy* than brass or string instruments. There was no difference in *airy/breathy* ratings between brass and strings.

### Extracting Audio Features

To investigate the relationships between semantic timbre categories and audio features, we used an updated version of the Timbre Toolbox. The Timbre Toolbox calculates spectral, temporal, and spectrotemporal audio features from an audio signal in Matlab (MATLAB, 2021). First, input representations of the signal are computed. Then, both scalar and time-series features are extracted from the different input representations. Lastly, the Timbre Toolbox calculates interquartile range (IQR) and median values of the time-series of spectral features computed on successive timeframes. These values represent the central tendency and variability of the audio features (Peeters et al., 2011). For this study, we used the STFT (Short-Time Fourier Transform), HARM (Harmonic), and TEE (Temporal Energy Envelope) input representations. The STFT is a spectrotemporal representation obtained using a sliding-window analysis over the audio signal. Then, the amplitude spectrum of the STFT is used as one of the representations to derive the audio features. HARM (sinusoidal harmonic model) is a harmonic representation that uses frame analysis to estimate the slowly varying amplitudes and frequencies of harmonic components of an estimated fundamental frequency. The TEE is a representation based on the temporal envelope derived from the amplitude of the analytic signal (Peeters et al., 2011).

Each stimulus was analyzed in the Timbre Toolbox to determine the median and IQR of audio features from the STFT and HARM representations, as well as values from the TEE representation. Timbre Toolbox’s standard settings were applied in these analyses. Given that several features can be derived from both the STFT and HARM representations, such overlapping features were taken exclusively from the STFT representation. All together, we used medians and IQRs of 22 spectral features and values of eight temporal features for a total of 52 initial input variables to the models (Table 3).

**TABLE 3:** Audio features extracted from Timbre Toolbox.

Audio features from Timbre Toolbox		
Representation	Feature	Description
STFT	Spectral centroid	Center of gravity of the spectrum
STFT	Spectral spread	Standard deviation of the spectrum around the mean
STFT	Spectral skewness	Asymmetry of the spectrum around the mean
STFT	Spectral kurtosis	Flatness of the spectrum around the mean
STFT	Spectral flatness	Ratio of the geometric and arithmetic means of the spectrum
STFT	Spectral crest	Ratio of the spectral maximum to the arithmetic spectral mean
STFT	Spectral slope	Linear regression over the spectral amplitude values
STFT	Spectral decrease	Average of slopes between F0 and 2nd to *k*th harmonic
STFT	Spectral roll-off	Frequency below which 95% of the signal energy is contained
STFT	Spectral variation	A measure of variability of the spectrum over time: correlation between spectra in successive time frames
STFT	Spectral flux	A measure of variability of the spectrum over time: Euclidean distance between spectra in successive time frames
HARM	F0	Fundamental frequency of a periodic sound
HARM	Harmonic spectral deviation	Deviation of the amplitudes of the partials from a smoothed spectral envelope
HARM	Tristimulus 1	Ratio of energy of the 1st harmonic to total energy
HARM	Tristimulus 2	Ratio of energy of the 2nd, 3rd, and 4th harmonics to total energy
HARM	Tristimulus 3	Ratio of energy of remaining harmonics (above 4th) to total energy
HARM	Harmonic odd-to-even ratio	Ratio of energy of odd harmonics to even harmonics
HARM	Inharmonicity	Degree to which frequencies of overtones depart from multiples of the fundamental frequency
HARM	Harmonic energy	Energy of the signal explained by stable partials
HARM	Noise energy	Energy of the signal not explained by stable partials
HARM	Noisiness	Ratio of noise energy to total energy
HARM	Harmonic-to-noise ratio	Ratio between periodic and non-periodic components of a signal
TEE	Attack time	Duration of the attack portion of the sound
TEE	Log attack time	Logarithm of the duration of the attack portion of the sound
TEE	Attack slope	Rate of change of energy over time in the attack portion
TEE	Decrease slope	Measure of the rate of decrease of the signal energy
TEE	Temporal centroid	Center of gravity of the energy envelope
TEE	Effective duration	Time during which energy envelope is above 40% (intended to reflect perceived duration)
TEE	Frequency of energy modulation	Frequency of the modulation of energy over the sustained portion of the sound as represented using a sinusoidal component
TEE	Amplitude of energy modulation	Amplitude of the modulation of energy over the sustained portion of the sound as represented using a sinusoidal component

*STFT, short-time Fourier transform; HARM, harmonic; TEE, temporal energy envelope. For further detail on how features are computed, see Kazazis et al. (2021).*

**FIGURE 4:**
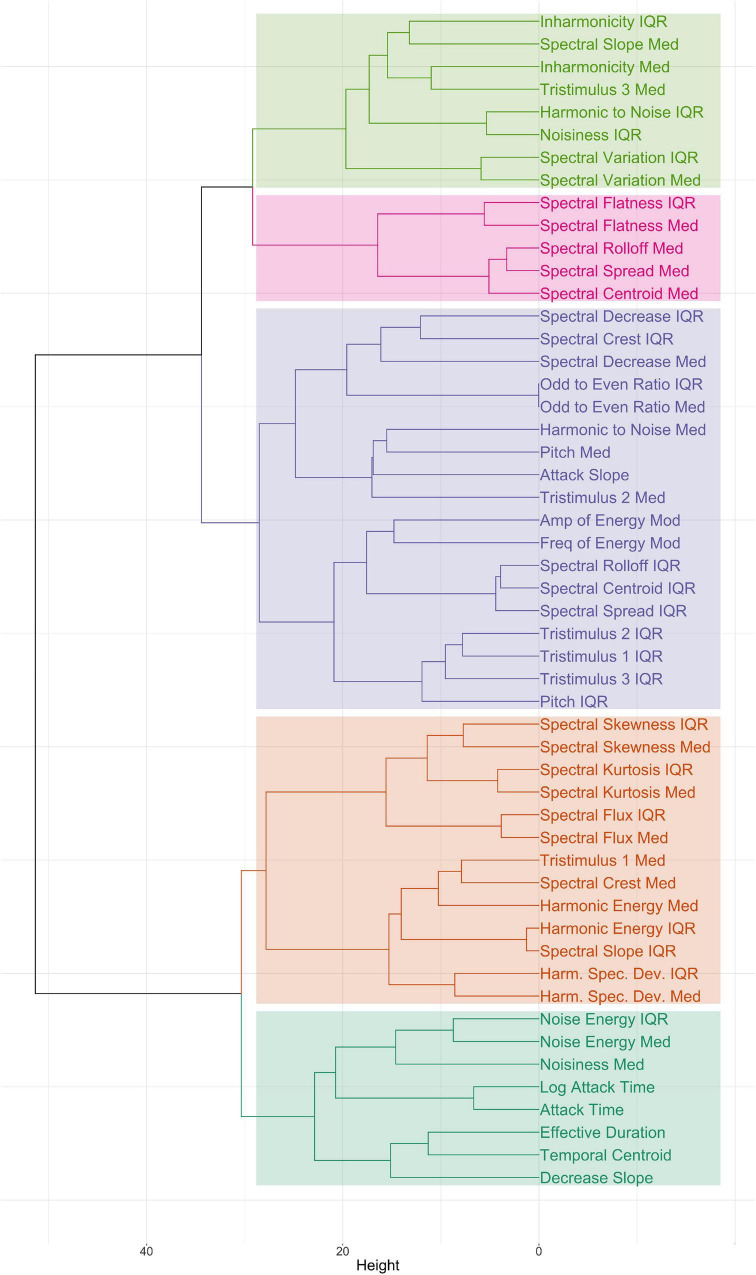
Dendrogram of hierarchical clustering of feature values across the stimulus set. Colors indicate groups of features organized by a five-cluster solution.

Prior to building the models, we performed an analysis of collinearity among descriptors across the stimulus set. As in previous literature (e.g., Peeters et al., 2011; McAdams et al., 2017), descriptors were multicollinear. A hierarchical cluster analysis was performed using Ward linkage with Euclidean distance; the dendrogram, which demonstrates the high multicollinearity, is shown in Figure 4. The overall Kaiser–Mayer–Olkin index was 0.70, and Bartlett’s test of sphericity was significant, suggesting that factor analysis would be appropriate for the data.

### Modeling

Exploratory modeling was carried out in order to investigate the relationships between audio features and semantic ratings. McAdams et al. (2017) found that a non-linear modeling approach was more successful than a linear approach in modeling affective ratings of musical instrument sounds. To assess whether this observation would generalize to semantic ratings in a different dataset, we also performed both linear and non-linear modeling. Scaled and centered values for the audio features extracted from the Timbre Toolbox were used to predict mean semantic ratings. Separate models were generated for each of the three semantic categories as well as for valence and exertion.

Considering the collinearity in the data evidenced by the hierarchical clustering shown in Figure 4, we chose to use partial least-squares regression (PLSR) as our linear modeling method, a supervised learning algorithm that takes a dimension-reduction approach by including a Principal Component Analysis process. Unlike principal component regression, however, PLSR takes both the predictor and outcome variables into account when building the linear model. This kind of statistical approach can handle data that exhibits multicollinearity and thus was appropriate for our dataset. Random forest regression was used as the non-linear method of analysis. A random forest (RF) is a supervised machine learning algorithm that uses an ensemble learning method, building multiple decision trees by randomly selecting observations and specific variables and then averaging the predictions from each tree (Biau and Scornet, 2016). Both types of models were built with the *caret* package (Kuhn, 2020) in R, version 4.0.5 (R Core Team, 2020).

In testing, we applied fivefold cross-validation to each model. The observations were divided into five subsets; the model was trained on four out of the five subsets and then its ability to predict the last remaining subset was estimated. The subsets were rotated to ensure that the training and prediction steps were applied to every combination of the subsets. Within each of the train-test subsets, models were trained using a 10-fold cross-validation repeated three times.

After comparing models using features from all three representations (52 features) with models using spectral features only (44), we found that the addition of temporal descriptors did not meaningfully improve the models in terms of measures of fit like *R*^2^ or root mean squared error (RMSE). This was the case even though the temporal features did not exhibit high levels of multicollinearity with features derived from the other two representations. Furthermore, temporal descriptors were not ranked highly in relative variable importance (RVI) in any of the models. Thus, we report and discuss the results for the models using the 44 spectral features from only the STFT and HARM representations in subsequent sections.

Average *R*^2^ is used as a measure of predictive relevance derived from the cross-validation procedure. Values for average RMSE and average *R*^2^ for models reported in Table 4 are averaged over the results of the fivefold cross-validation.

**TABLE 4:** Summary of models predicting mean ratings from audio features.

Rating	Model type	*R* ^2^	Average *R*^2^	Average RMSE
*Raspy/Grainy/Rough*	RF	0.82	0.78	0.47
	PLSR	0.64	0.56	0.70
*Harsh/Noisy*	RF	0.56	0.54	0.69
	PLSR	0.43	0.28	0.93
*Airy/Breathy*	RF	0.45	0.43	0.78
	PLSR	0.36	0.29	0.89
Exertion	RF	0.34	0.32	0.84
	PLSR	0.14	0.09	1.08
Valence	RF	0.68	0.67	0.59
	PLSR	0.56	0.52	0.72

*R^2^ values are calculated from models run on the entire dataset (trained using 10-fold cross validation repeated three times). Average R^2^ values are a measure of predictive relevance representing test R^2^ averages across the fivefold cross validation process. RMSE is also averaged across the fivefold.*

In all five models, the random forest analysis resulted in better fit than did partial least-squares regression, with an average improvement in *R*^2^ of 0.2 (20% explained variance) for the random forest models. The model predicting ratings of *raspy/grainy/rough* was the most successful, whereas the *exertion* model was the least successful.

We calculated RVI using the *varImp* function in the *caret* package (Kuhn, 2020). RVI values are reported in Tables 5, 6. RVI for the PLSR is based on the weighted sums of the absolute regression coefficients. The weights are a function of the reduction of the sums of squares across the number of PLS components and are computed separately for each outcome (Kuhn, 2020). To compute RVI for the RF, the mean squared error is recorded on the out-of-bag portion of the data (data points not selected randomly for a given sample) and after permuting each predictor variable. Differences between these values are averaged across all trees and normalized by the standard deviation of the differences (Liaw and Wiener, 2002).

**TABLE 5:** Top 10 important variables and their respective relative variable importance values for each semantic category using partial least-squares regression.

*Airy/breathy*		*Harsh/noisy*		*Raspy/grainy/rough*		Valence		Exertion	
Feature	RVI	Feature	RVI	Feature	RVI	Feature	RVI	Feature	RVI
Harm Spec Dev IQR	100	Spectral Decrease Med	100	HNR Med	100	HNR Med	100	Tristimulus 3 Med	100
Harm Spec Dev Med	72.27	Spectral Centroid Med	64.79	Noisiness Med	92.05	Noisiness Med	98.86	Spectral Centroid IQR	93.03
Spectral Roll-Off Med	68.76	F0 Med	54.92	Spectral Variation IQR	74.63	Inharmonicity Med	82.78	Spectral Roll-Off IQR	84.34
Spectral Spread Med	64.50	Spectral Spread Med	50.88	Harmonic Energy Med	73.24	Tristimulus 3 Med	82.68	Tristimulus 1 Med	77.77
Spectral Centroid Med	62.17	Spectral Roll-Off Med	50.03	Inharmonicity Med	73.17	Spectral Crest Med	77.59	Inharmonicity Med	74.58
Spectral Flux IQR	58.61	Spectral Variation IQR	46.63	F0 Med	72.26	F0 Med	75.73	Harmonic Energy Med	70.78
Spectral Slope IQR	58.24	Harm Spec Dev IQR	40.62	Spectral Slope Med	70.63	Harm Spec Dev Med	73.49	Spectral Spread IQR	69.89
Tristimulus 1 Med	56.17	Harm Spec Dev Med	39.97	Spectral Crest Med	66.84	Harm Spec Dev IQR	74.19	Spectral Slope Med	68.82
Spectral Flux Med	52.86	HNR Med	31.52	Tristimulus 3 Med	63.57	Spectral Variation IQR	73.00	Harm Spec Dev Med	61.42
Spectral Skewness Med	48.44	Spectral Decrease IQR	31.36	Spectral Variation Med	59.95	Harmonic Energy Med	68.10	Spectral Crest Med	58.98

**TABLE 6:** Top 10 important variables and their respective relative variable importance values for each semantic category using random forest regression.

*Airy/breathy*		*Harsh/noisy*		*Raspy/grainy/rough*		Valence		Exertion	
Feature	RVI	Feature	RVI	Feature	RVI	Feature	RVI	Feature	RVI
Odd:Even Ratio Med	100	Spectral Decrease Med	100	HNR Med	100	Inharmonicity IQR	100	Noisiness IQR	100
Odd:Even Ratio IQR	72.34	Spectral Spread Med	87.68	Inharmonicity IQR	62.11	HNR Med	55.98	F0	83.62
Harm Spec Dev IQR	71.73	Spectral Roll-Off Med	72.04	Spectral Variation Med	54.34	Tristimulus 3 Med	19.59	HNR IQR	77.18
Spectral Roll-Off Med	49.60	Spectral Centroid Med	71.21	Noisiness Med	40.84	Odd:Even Ratio Med	14.55	Noise Energy Med	72.15
Spectral Flux IQR	40.98	Spectral Spread IQR	62.49	Spectral Variation IQR	39.43	Noisiness Med	13.73	Tristimulus 1 Med	69.95
Spectral Spread Med	30.97	Spectral Variation IQR	37.58	Tristimulus 3 Med	21.00	Spectral Variation Med	8.77	Tristimulus 3 Med	64.35
Spectral Centroid Med	29.13	Spectral Flatness IQR	31.09	Inharmonicity Med	18.98	Inharmonicity Med	6.63	Harmonic Energy Med	62.72
Spectral Variation IQR	27.13	Spectral Variation Med	26.13	F0 Med	18.81	Harm. Spectral Deviation IQR	5.97	Inharmonicity Med	60.57
Spectral Skewness IQR	19.02	Spectral Flatness Med	24.44	Harmonic Energy Med	5.11	F0 Median	5.30	Spectral Variation Med	55.64
Tristimulus 1 IQR	16.67	Noisiness IQR	24.24	Tristimulus 1 Med	3.13	Spectral Variation IQR	4.12	Spectral Slope IQR	41.25

The median harmonic-to-noise ratio (HNR) was the most important variable in predicting ratings of *raspy/grainy/rough*; other features related to the relative role of harmonic and stable partials, including inharmonicity and noisiness, were given high RVI values. Noisiness median and HNR median were nearly perfectly negatively correlated with each other, where increased ratings of *raspy/grainy/rough* were associated with increased noisiness and decreased HNR. However, the relationship between these features and the ratings are non-linear in part because these features are bimodally distributed across stimuli. Inharmonicity IQR increases with ratings of *raspy/grainy/rough*, though the relationship appears to be non-linear, perhaps exponential. Although these types of features, which have been associated with noisiness in general, were important for the *raspy/grainy/rough* model, they were unexpectedly of relatively little importance in predicting ratings of *harsh/noisy* and *airy/breathy*.

Spectral decrease median was the most important feature for predicting *harsh/noisy*, with a moderate negative correlation. The top 10 most important features, including spread, roll-off, and centroid, each have positive relationships with *harsh/noisy* ratings. Many of the flute and recorder sounds demonstrated low spread, roll-off, and centroid and were rated low on *harsh/noisy*; in contrast, the tenor saxophone screams provide examples of stimuli demonstrating high values on these features that were also rated as high on *harsh/noisy*.

Harmonic odd-to-even ratio median and IQR were the most important features for *airy/breathy*, as well as the harmonic spectral deviation IQR. Flute and recorder stimuli in general demonstrated significantly higher odd-to-even ratio medians and IQR values. Although there is a positive relationship between these features and ratings without members of the flute family, it is rather weak. Harmonic spectral deviation represents the deviation of the amplitudes of the partials from a smoothed energy envelope—that IQR is important here suggests that it is the amount of variation in this deviation that may contribute to the perception of *airy/breathy*. Spectral features, including roll-off, flux, spread, and centroid, were also placed among the top ten most important features in predicting *airy/breathy* ratings.

Spectral variation IQR, which can be thought of as the variation of the variation in a spectrum over time, was in the top 10 important features predictive of ratings for all three semantic categories. This illustrates a way in which temporal irregularity is a central feature for all three categories. However, while the relationship between this feature and both *raspy/grainy/rough* and *harsh/noisy* is clearly positive in nature, the relationship with *airy/breathy* is less obvious and is weaker. For example, both the bass flute playing with various unpitched air sounds and all types of flutes playing with flutter-tongue were rated as highly *airy/breathy*; however, the unpitched air sounds stimuli had relatively high spectral variation IQR, whereas the flutter-tonguing flutes had relatively very low spectral variation IQR.

Although there was some overlap in features with high RVI values for the three semantic categories, patterns of variable importance were distinct for each semantic category. Particularly among the RF models, features ranking especially high in relative importance were often unique to one of the three semantic categories, though some important features overlapped between categories. This suggests that specific combinations of features may be important for the perception of varying semantic information.

The most relevant variables in predicting valence included inharmonicity IQR and the HNR median, which was also true of the *raspy/grainy/rough* model. This overlap is cogent in that ratings for *raspy/grainy/rough* were strongly, negatively correlated with valence. In fact, eight of the top 10 most important variables for valence overlap with the top 10 for *raspy/grainy/rough*, with the exception of two variables which were among the top three for *airy/breathy*.

The majority of important variables in our random forest model predicting perceived exertion were from the harmonic representation, including noisiness IQR, F0 median, and HNR median. Sounds perceived as requiring more exertion tended to have higher noisiness, lower HNR median, and lower median F0. The overlapping important features relevant for perceived exertion and *raspy/grainy/rough* as well as *harsh/noisy* further demonstrate the link between these semantic categories and the perception of exertion.

## Discussion

### Listener Ratings

#### Valence

Our predictions for valence correlations were confirmed: valence was positively correlated with *airy/breathy* and negatively correlated with *raspy/grainy/rough* and *harsh/noisy*. This positive correlation with *airy/breathy* is consistent with Reymore and Huron’s (2020) suggestion that although *airy/breathy* instrumental timbres may share audio feature markers with other nominally “noisy” categories, the quale often carries a positive connotation due to evocations of closeness or intimacy, in parallel with breathy vocal timbres.

In Wallmark et al. (2018), participants rated sounds on a valence scale labeled as *like-dislike* and on noisiness. These scales are comparable to the valence and *harsh/noisy* scales in the current experiment. Our results converge with theirs in that both studies observed a moderately strong negative correlation of noisiness with valence: Wallmark’s correlation in Experiment 1 (single-note stimuli) was *r* = –0.55 and in Experiment 2 (polyphonic timbres) was *r* = –0.63, values which are comparable to our observed correlation of *r* = –0.61.

Contrary to our expectations, *raspy/grainy/rough* was more strongly negatively correlated with valence than was *harsh/noisy*. In examining the estimated marginal means for the linear mixed models reported in the following section, we noted that *raspy/grainy/rough* had a negative relationship with register (lower pitches are more *raspy/grainy/rough*), whereas *harsh/noisy* demonstrated an opposite tendency (i.e., higher pitches are more *harsh/noisy*). Yet, valence and *harsh/noisy* both demonstrated positive relationships with register. This confluence of observations may explain the relative strengths of the two semantic associations with valence: although both are ultimately negatively correlated with valence, the relationship of *harsh/noisy* to valence may be tempered by a tendency for higher pitches to be more positively valenced.

#### Exertion

As predicted, we found both *raspy/grainy/rough* and *harsh/noisy* to be positively correlated with exertion, but no significant correlation was observed between *airy/breathy* and exertion. Wallmark also observed a correlation between perceived exertion and noisiness, *r* = 0.55 in Experiment 1, *r* = 0.87 in Experiment 2. Our observed correlation, *r* = 0.46, was lower. Note that the range of correlations observed in these three experiments is much larger than that for valence, suggesting that valence/noisiness trends may be relatively more consistent in strength among stimulus sets than valence/exertion trends.

The negative trend between valence and exertion observed in our study, *r* = –0.48, was also observed in Wallmark et al. (2018). In that paper, Experiment 1 yielded a correlation of *r* = –0.17 (non-significant, likely due to sample size), and Experiment 2 resulted in a significant correlation of *r* = –0.67. Although McAdams et al. (2017) did not measure perceived exertion directly, participants rated stimuli on a tension arousal scale (tension-relaxation). Consistent with both the current results and those of Wallmark et al. (2018), they found that valence was correlated with tension arousal, such that more negatively valenced sounds were also rated as more tense, *r* = 0.46, a similar strength rating to that observed in the current study. In general, our stimulus set is more similar to that of McAdams et al. (2017), than to either set in Wallmark et al. (2018). This observation, along with the wide range of observed correlation values across these experiments, suggests that the strength of the valence/exertion relationship may also vary with stimulus set.

#### Semantic Scales

Correlations among the three semantic scales suggest that these semantic timbre categories are related, but also that they are distinct. *Airy/breathy* did not significantly correlate with *raspy/grainy/rough* but did correlate moderately negatively with *harsh/noisy*. And whereas *raspy/grainy/rough* correlated positively with *harsh/noisy*, as might be expected semantically, the strength of this correlation is still only moderate. These distinctions may be important in various musical contexts, such as when composers are choosing or synthesizing timbres with specific semantic connotations. More refined understanding of meaningful timbre categories can also benefit tasks such as predicting semantics or affect from audio features, with applications in music information retrieval and audio branding.

### Effects of Stimulus Properties on Semantic Ratings

Our results illustrate several connections between general stimulus properties and semantic associations. Linear mixed effect models predicting semantic ratings from the factors of instrument family, register, and extended technique demonstrated several significant relationships. Register and extended technique were both significant for ratings of valence, exertion, *harsh/noisy*, and *raspy/grainy/rough* ratings; instrument family was significant for *airy/breathy* ratings.

In particular, the findings highlight the complexities of pitch register on semantic associations. Although semantically, *raspy/grainy/rough* and *harsh/noisy* seem to be closely related, we observed that mean ratings of *raspy/grainy/rough* decrease with increasing register, but mean ratings of *harsh/noisy* increase with increasing register. Thus, register may be a key factor in differentiating these two qualia. This observation is relevant to Reymore and Huron’s (2020) timbre qualia model. Recall that the original *harsh/noisy* dimension from that model includes not two, but three terms—*shrill, harsh*, and *noisy*. Reymore (in press) found that *shrill/harsh/noisy* was both a significant predictor and among the most important variables for prediction of register in both the oboe and French horn. Similarly, Reymore et al. (2021) found that across eight orchestral instruments, *shrill/harsh/noisy* increased with increasing register. However, because of the inclusion of the pitch-related word *shrill*, those studies remained unclear on the extent to which those relationships were being driven by the connotations of the word *shrill* as compared to the other two terms (*harsh, noisy*). In the current study, which did not include the word *shrill*, we nevertheless observed that *harsh/noisy* is associated with higher pitch, consistent with the inclusion of *shrill* in the original dimension. In Reymore (in press), *raspy/grainy/rough* was also a significant predictor for register in both oboe and horn, though it scored relatively low in variable importance. This trend of decreasing ratings of *raspy/grainy/rough* with increasing register was also observed in Reymore et al. (2021).

In the current study, *airy/breathy* did not demonstrate a significant trend with register; this is consistent with modeling in Reymore (in press), in which *airy/breathy* was not a significant predictor of register for either oboe or horn and did not rank highly in either random forest model. Reymore et al. (2021) did observe a significant effect of register on *airy/breathy* ratings, though the difference appears only to be between the low and middle registers, and the marginal *R*^2^ value for the full model (with instrument and register as predictors) was low. One possible explanation could be that, semantically speaking, *airy* may be more likely to be used to describe higher pitched sounds, whereas *breathy* may be considered more apt in describing lower pitched sounds. If this were the case, we would not necessarily expect to see a relationship of the *airy/breathy* quale with register, as it includes both terms.

Extended technique, as a categorical variable, was a significant predictor for *raspy/grainy/rough*, *harsh/noisy*, and exertion (sounds produced using extended techniques were associated with higher ratings on these scales) as well as valence (sounds produced using extended techniques were associated with lower valence ratings). This result must be interpreted with caution, as the inclusion of specific techniques in our stimulus set was guided by the need to sample sounds at the extreme ends of the scales of interest rather than to sample evenly across techniques. Thus, we cannot generalize these results to all extended techniques. The analysis suggests that certain extended techniques affect semantic associations, but we do not have enough information to draw conclusions about the relationships between particular techniques and particular semantic associations. However, the results suggest that investigation of such relationships may be productive in future research, revealing semantic associations with various types of techniques across instruments. Such results would be useful in consideration of orchestration and music analysis.

*Airy/breathy* was the only scale to demonstrate a significant relationship with instrument family. This relationship was likely driven in part by the number of stimuli played on different types of flutes and recorders. During stimulus selection, the authors sought to choose a range of types of stimuli representing the *airy/breathy* quality; however, it was apparent that among orchestral sounds, flutes and recorders generally provide the best examples of the *airy/breathy* quale. Other instruments receiving relatively higher ratings on *airy/breathy* were also often woodwinds, including samples from the clarinet, bass clarinet, and contrabassoon. Among stimuli receiving an average rating of over 4 on this scale, only a few were from other instrument families, including two samples of tuba, one of trombone, and two double bass samples with extended techniques. However, after ordering stimuli by mean ratings of *airy/breathy*, the first non-woodwind stimulus does not appear until the 22nd item on the list.

### Modeling Semantics With Audio Features

Although the three semantic categories of interest demonstrate different semantic properties, as evidenced by their varying correlations with valence and effort, there is also apparent semantic similarity between *raspy/grainy/rough* and *harsh/noisy*, which are moderately correlated and are associated with both increased exertion and decreased valence. Although many of the relationships we observed between ratings and features are non-linear, it is evident from studying the Spearman correlations that some of the features share directional tendencies between pairs of categories or even among all three categories. For example, higher ratings on all three semantic categories are associated with increased noisiness median and IQR, spectral flux median, and F0 IQR. The strengths of these relationships vary among categories: for example, the monotonic positive relationship with noisiness median for *airy/breathy* is 0.19, for *harsh/noisy* is 0.27, and for *raspy/grainy/rough* is 0.81. The modeling in this paper was aimed at uncovering which underlying audio features best explain and distinguish these three semantically close, yet distinct, categories.

#### Semantic Categories

Partial least-squares regression models and random forest models were built to predict mean semantic ratings from extracted audio features. These models were most successful in predicting ratings of *raspy/grainy/rough*; models predicting *harsh/noisy* and *airy/breathy* explained less variance but were still moderately successful (Table 4). McAdams et al. (2017) found that a non-linear approach explained more variance in the data than a linear approach when modeling affective qualities using audio features. We also observed an advantage for the non-linear method, as random forest models consistently demonstrated better fit with lower RMSE values than the linear PLSR models. Because random forest regression offered the more successful models, the current discussion of results focuses on the random forest models unless otherwise noted.

In general, we found both spectral and spectrotemporal features to be relevant for modeling the target semantic ratings, and it is notable that the addition of the eight features from the temporal representation did not improve fit for any of the models. We found that the IQR values of several features—which can be considered spectrotemporal in that they describe spectral variation over time—contributed to both linear and non-linear models, including the *harsh/noisy* models. For the three semantic categories, IQR values contributed more to the non-linear than the linear models, based on comparisons of both proportion of IQR values in the top ten and their total relative importance values. Models in which IQR values were included in the top three most important descriptors included *airy/breathy* (both linear and non-linear), *raspy/grainy/rough* (both linear and non-linear), *valence* (both linear and non-linear), and *exertion* (non-linear only). Notably, although RVI values for *harsh/noisy* did include IQRs, these were not among the top three descriptors as they were for most other models. In their study of the perceptual dimensions of timbre, Elliott et al. (2013) concluded that a principal component, which they labeled “noisy, small instrument, unpleasant,” either did not depend on spectrotemporal modulations (as measured through the Modulation Power Spectrum) or did so in a non-linear way. Although we did observe connections between spectrotemporal features and *harsh/noisy*, the role of these features was less important for this category in comparison to others.

One method of comparing feature importance among the three semantic categories and working toward an understanding of how they may be distinguished is to choose a minimum importance value in order to define what constitutes a “relevant” feature. Relevant features can then be compared across models. For example, spectral variation IQR is the only feature with an RVI value over 25 for all three semantic categories, suggesting that it is at least moderately relevant for all three categories.

In this manner, we can identify which features are uniquely relevant to each semantic category, where relevance is operationalized as describing features with RVI values over 25. Because the measure ranks relative importance, the choice of a minimum importance threshold is necessarily somewhat arbitrary: 25 was chosen for the purpose of this discussion based on the distribution of RVI values in the random forest models and because this threshold resulted in a reasonable number of features for consideration in discussion. Definitions of relevance in similar interpretations could be adjusted depending on the goals of the interpretation and/or factors such as how many features are practical for consideration for a given application.

With this definition in mind, uniquely relevant features for *raspy/grainy/rough* include the HNR median, inharmonicity IQR, and noisiness median. Of these features, median HNR and median noisiness were strongly negatively correlated in the dataset, *r*(154) = –0.96, *p* < 0.001. For *harsh/noisy*, uniquely relevant features include the spectral decrease median, spectral spread IQR, and spectral flatness IQR. Unique features for *airy/breathy* include the harmonic odd-to-even ratio (median and IQR, which are correlated at *r* = 0.99), harmonic spectral deviation IQR, and spectral flux IQR.

The spectral variation median was relevant for both *raspy/grainy/rough* and *harsh/noisy*. *Harsh/noisy* and *airy/breathy* also shared relevant features—spectral roll-off median, spectral spread median, and spectral centroid median; these three features were strongly correlated across our stimulus set (roll-off/spread, *r* = 0.97; roll-off/centroid, *r* = 0.95, centroid/spread, *r* = 0.91).

**FIGURE 5:**
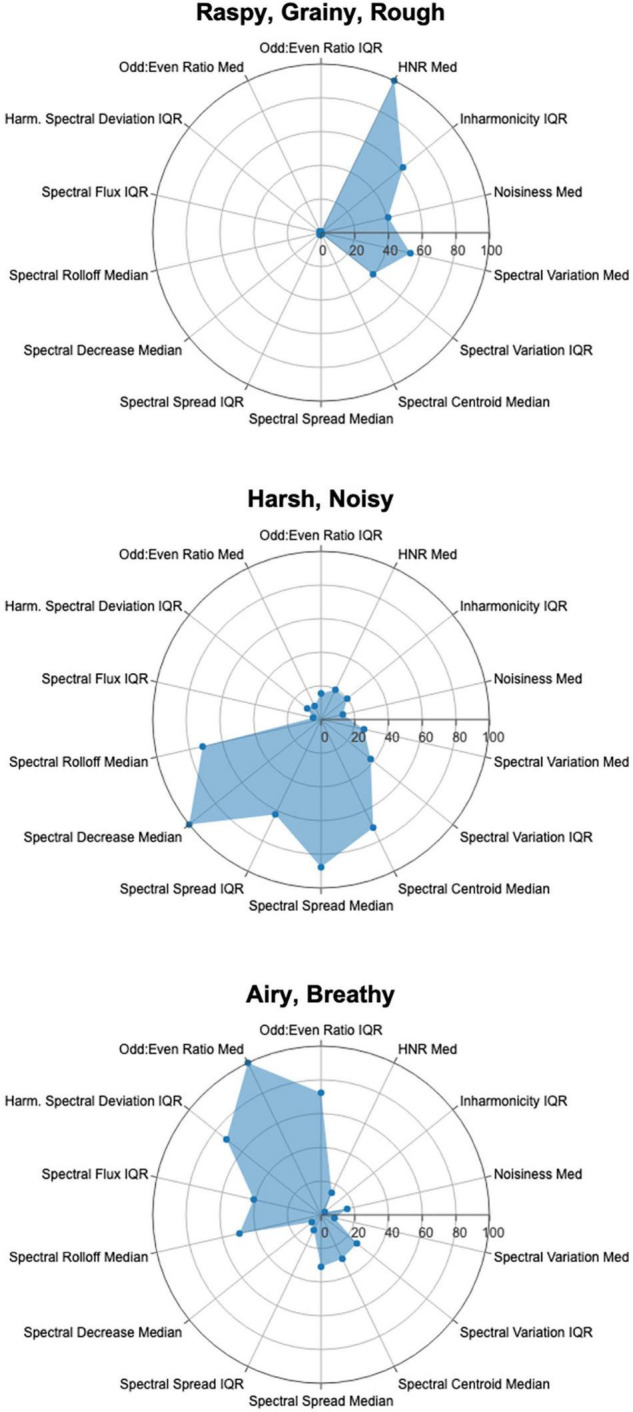
Radar plots of 14 most important features across the three semantic categories in the random forest models. Radius represents relative variable importance values.

Relative variable importance values for the 14 most important features across categories from the random forest models are illustrated in the radar plots of Figure 5. The radius represents RVI; features are listed in the same order around the circles for all three plots in order to facilitate visual comparisons of semantic categories. Each of the three categories yields a visually distinctive plot, demonstrating that although some important features overlap across categories, the categories exhibit separable audio feature profiles. These distinct audio profiles offer evidence that different combinations of “noise”-related audio features correspond to different semantic concepts.

Wallmark et al. (2018) found positive relationships between perceived noisiness and high frequency energy, spectral centroid, inharmonicity, and spectral flatness in both experiments, though not all were significant. We observed significant positive correlations between ratings of *harsh/noisy* and medians of spectral centroid (0.46, *p* < 0.001) and spectral flatness (0.12, *p* < 0.001). *Harsh/noisy* was positively related to both spectral slope and Tristimulus 3 values (Pollard and Jansson, 1982), which bear some relation to the high-frequency energy feature. Yet, we did observe a significant, moderately strong relationship between inharmonicity and *raspy/grainy/rough* (0.40), and inharmonicity related values were important in both linear and non-linear models. These observations further support the claim that *harsh/noisy* and *raspy/grainy/rough* descriptors are both semantically and acoustically distinguishable, and that inharmonicity is more closely related with *raspy/grainy/rough*. Conversely, spectral centroid median appears to be closely related to *harsh/noisy* but not to *raspy/grainy/rough*.

#### Valence

The most relevant variables in predicting valence included inharmonicity IQR and the HNR median. The results of McAdams et al. (2017) can only be compared indirectly to the current results, as their study did not examine features from the Harmonic representation in the Timbre Toolbox and included some of the features from the temporal representation. Although we used the same linear method to model results, the non-linear modeling approach was different (random forest vs. neural net), and variable importance was calculated with a different method. Overall, there appears to be relatively little agreement, likely in part because of the consistently strong role of harmonic descriptors in our models, which were not included in the (McAdams et al., 2017) paper. For non-linear models in both studies, spectral variation median and IQR were marked as important for predicting valence. However, all other variables among the top 10 most important variables for valence in our model were from the harmonic representation.

Results from Eerola et al. (2012), in which valence was predicted from audio features of stimuli at the same frequency, can also be considered in relation to our results. They used a reduced set of audio features chosen through principal component analysis and observed linear relationships. Three audio features—ratio of high- to low-frequency energy, envelope centroid, and spectral skewness—explained 60% of the variance in valence ratings. We did not include ratio of high- to low-frequency energy as a descriptor, but both linear and non-linear valence models in our experiment ranked the tristimulus 3 median—the ratio of the relative weight of all harmonics above the fourth—as highly relevant for valence. A larger tristimulus 3 median indicates more high-frequency energy relative to the fundamental; our value was negatively correlated with valence, indicating that more positive valence was associated with less high-frequency energy. Although these two measures (ratio of high- to low-frequency and tristimulus 3 median) are not equivalent, they are both relative measures of higher-frequency energy, and so in this respect, our results converge. Similarly, we also observed that more negative spectral slope median (i.e., the audio signal trails off toward higher frequencies more quickly, suggesting less high-frequency energy) is associated with more positive valence (*r* = –0.50). Finally, we only observed weak linear relationships between valence and the other two features identified by Eerola et al. (2012)—temporal centroid and spectral skewness—neither of which was significant. Furthermore, spectral skewness did not serve an important role in the non-linear model.

We ultimately did not include temporal descriptors in our final models, given that they did not improve model fit. Thus, it may be possible that the temporal descriptors were providing redundant information that was already somehow captured by correlations with harmonic descriptors. Yet, at least from the surface, this does not appear to be the case, as the temporal descriptors in general showed mostly weak and some moderate correlations with the rest of the descriptors (both Pearson’s and Spearman’s correlations were considered).

#### Exertion

The majority of important variables in our random forest model predicting exertion were from the harmonic representation, including noisiness IQR, F0 median, and harmonic to noise energy ratio median. The spectral variation median appears to be the only overlapping factor of any importance between the tension arousal models from McAdams et al. (2017) and the exertion models in the current study. The high importance of spectral components in both linear and non-linear models of exertion in the current study (including F0 median, tristimulus 1 median, tristimulus 3 median) differs relative to the other models, as the relationship of exertion with these features was comparatively weaker, and it also demonstrated concavity. However, we can consider that overall, exertion was the least successful of the five models—so it seems that even though the model was able to capitalize on pitch-related features, this prominence may be highlighted because it was unable to capitalize on many of the other features which served the other four models better. However, Wallmark et al. (2018) did observe strong, significant correlation between high-frequency energy and exertion ratings (0.71) in single-note stimuli; this may be reflected in our findings through the positive correlations between exertion and both tristimulus 3 median (0.42) and spectral slope (0.30).

Zacharakis and Pastiadis (2021) found that time-varying inharmonicity affects continuous responses of tension during 30-s synthetic complex tones. We found inharmonicity median for single-note stimuli to be among the top 10 most important features for predicting exertion ratings in both linear and non-linear models. The current results and those of Zacharakis and Pastiadis (2021) suggest that inharmonicity is strongly associated with perceived exertion and felt tension. Even if these two ratings are measuring different concepts, Wallmark’s motor-mimetic account of timbre perception would suggest that the two would be related, as ecologically, the experience of physical exertion is coupled with felt physical tension. Thus, sounds that connote physical exertion may activate motor areas of the brain and thus be cognitively associated with tension (see Wallmark et al., 2018 for a review). We could also speculate that such brain activity might somehow contribute to the experience of felt tension, though such a claim certainly requires further research.

## Conclusion

This study examined the association between combinations of audio features and perceived semantic content pertaining to noise in instrumental sounds, specifically *airy/breathy*, *raspy/grainy/rough*, *harsh/noisy*, valence, and perceived exertion. Together, our results suggest that listeners make distinctions between the semantic categories *harsh/noisy*, *raspy/grainy/rough*, and *airy/breathy*. Even though individual variance in response is evident, our participants were generally consistent in how they used these terms to rate short, single-note instrumental stimuli across a range of instruments, registers, and playing techniques. This offers converging evidence that listeners can provide generally consistent ratings of affect (e.g., Eerola et al., 2012; McAdams et al., 2017) and finely grained semantic categories (Reymore et al., 2021; Reymore, in press). In addition, the results of this experiment with respect to the relationship between valence and perceived exertion as related to noisy timbre provide converging evidence consistent with Wallmark’s (2014; Wallmark et al., 2018) embodied account of timbre.

In our analyses, we found that multiple semantic categories may be signified by shared auditory cues, such as spectral variation IQR, which was moderately important for all three categories. However, each semantic category also appears to some extent to have an “auditory fingerprint,” to the extent that both linear and non-linear models use distinct sets of audio features in predicting semantic ratings. The predictive success of these models for different semantic categories varied greatly; for example, average *R*^2^ across cross-validation folds for *raspy/grainy/rough* was 0.78, whereas it was 0.43 for *airy/breathy*. Other factors are likely at play in these semantic categories, including previous knowledge or understanding of how a sound may have been produced. For example, participants may have been more likely to rate a sound higher on *airy/breathy* if they recognized an instrument as some type of flute. Overall, feature models were least successful in predicting exertion and most successful in predicting ratings of *raspy/grainy/rough*.

Work on emotional meaning in sound and music has considered an adaptation of Brunswick’s lens model as a potential theoretical explanation for how listeners interpret and label affect. The lens model was adapted to vocal expressions by Scherer (1978) and has since been used to describe how expressed emotions are communicated from performer to listener (e.g., Juslin and Laukka, 2003). The model suggests that cues operate probabilistically, which helps cut through noise in communication due to factors such as individual differences and contextual effects. Eerola et al. (2013) discuss the ways in which their results are consistent with this model. A similar scenario might apply to the use of timbre semantic categories to describe sound. Although semantic descriptors are subject to some of the same variation of affective evaluations, including cultural and individual differences, semantic descriptors, at least in a Western context, are likely also subject to additional differences in how individuals interpret meanings of words in relation to sound in general, related to observations about the difficulty of describing timbre and the lack of an established lexicon.

Our results clarify relationships between low-level audio features and noise-related timbre semantic categories, contributing to efforts to bridge understandings of timbre in the fields of music cognition and music informatics. The findings from this study have the potential to influence approaches to timbre semantics, composition, and phonetics, with practical applications in audio branding and music pedagogy. This experiment also provides a basis for work on the underpinnings of semantic timbre categories. To more precisely understand how the audio features identified in this experiment contribute to the target noise-related semantic categories, findings could be used to synthesize timbres that systematically vary features of interest, with the goal of producing sounds that listeners will perceive as *harsh/noisy*, *airy/breathy*, etc. Future research can apply these results in musical and compositional contexts to validate the relationship between audio features and timbre semantic content and can furthermore examine how that relationship interacts with the relationships between dimensions of musical experience, such as tension arousal, and musical dimensions such as pitch, dynamics, harmony, and tempo. Furthermore, the methods described in this paper can be used to build feature profiles of other semantic categories beyond those related to noise.

## Data Availability Statement

The original contributions presented in the study are included in the article/Supplementary Material, further inquiries can be directed to the corresponding authors.

## Ethics Statement

The studies involving human participants were reviewed and approved by McGill University Research Ethics Board II. The patients/participants provided their written informed consent to participate in this study.

## Author Contributions

LR conceptualized the study. LR, EB-L, SM, and BS designed the experiment. BS created the experiment interface and collected the data. LR analyzed data. LR, EB-L, and SM interpreted the results. EB-L and LR wrote the manuscript. All authors contributed to editing and revisions.

## Conflict of Interest

The authors declare that the research was conducted in the absence of any commercial or financial relationships that could be construed as a potential conflict of interest.

## Publisher’s Note

All claims expressed in this article are solely those of the authors and do not necessarily represent those of their affiliated organizations, or those of the publisher, the editors and the reviewers. Any product that may be evaluated in this article, or claim that may be made by its manufacturer, is not guaranteed or endorsed by the publisher.
