# Supplementary material for: Modeling Noise-Related Timbre Semantic Categories of Orchestral Instrument Sounds With Audio Features, Pitch Register, and Instrument Family
Source: Front Psychol. 2022 Apr 1;13:796422. doi: 10.3389/fpsyg.2022.796422 (PMC9010607; doi:10.3389/fpsyg.2022.796422)
Supplement: Supplementary file 1 [file Data_Sheet_1.PDF]

Supplementary Material

Modeling noise-related timbre semantic categories of orchestral instrument sounds with audio features, pitch register, and instrument family

Lindsey Reymore*, Emmanuelle Beauvais-Lacasse, Bennett Smith, Stephen McAdams

*** Correspondence:** Lindsey Reymore, lindsey.reymore@mail.mcgill.ca

Table S1. Families, instruments, pitch registers, techniques (traditional v. extended), and databases of the stimulus set.

Instrument Family	Instrument	Technique	Pitch Register	Database
Brass	French horn	Traditional	2, 3, 4, 5	VSL
		Extended	3, 4, 5	VSL
		Traditional (brassy, *f*)	3, 4, 5	VSL
	Trumpet	Traditional	4, 5, 6	VSL
		Extended	4, 5, 6	VSL
		Extended	3, 3, 3, 3, 3, 4, 4	contimbre
	Bass Trumpet	Traditional	3	VSL
		Extended	3	VSL
	Tenor trombone	Traditional	3, 4, 5	VSL
		Extended	3, 4	VSL
		Extended	2, 2, 3, 3, 4, 4, 4	contimbre
	Bass trombone	Traditional	2, 3	VSL
	Contrabass trombone	Traditional	2, 3	VSL
		Extended	2, 3	VSL
	Tuba	Traditional	2, 3, 4	VSL
		Extended	3, 4	VSL
Woodwind	Flute	Traditional	4, 5, 6	VSL
		Extended	4, 5, 6	VSL
	Alto flute	Traditional	6	VSL
		Extended	4, 6	VSL
	Bass flute	Extended	3, 4	MUMS
		Extended	3, 4, 4	contimbre
	Pan flute	Traditional	3, 4, 5	MUMS
	Soprano recorder	Traditional	5, 6	MUMS
	Tenor recorder	Traditional	5, 6	MUMS
	Bass recorder	Traditional	2, 3	MUMS
	Oboe	Traditional	4, 5, 6	VSL
		Extended	4, 5, 6	VSL
	English horn	Traditional	4, 5	VSL

		Extended	4, 5	VSL
	Soprano crumhorn	Traditional	4, 5	MUMS
	Alto crumhorn	Traditional	5	MUMS
	Tenor crumhorn	Traditional	4, 5	MUMS
	Bass crumhorn	Traditional	3	MUMS
	Alto saxophone	Traditional	5, 6	MUMS
		Extended	6	MUMS
	Tenor saxophone	Traditional	3, 5	MUMS
		Extended	3, 4, 5, 6	MUMS
	Baritone saxophone	Traditional	2, 3	MUMS
	Tubax	Extended	2	contimbre
	Clarinet	Traditional	5	VSL
		Extended	5	VSL
	Bass clarinet	Traditional	2, 3	VSL
	Bassoon	Traditional	2, 3	VSL
		Extended	2, 3	VSL
	Contrabassoon	Traditional	2, 3	VSL
		Extended	2, 3	VSL
String	Violin	Extended	5, 5, 5, 5	contimbre
	Viola	Traditional	3, 4, 5	MUMS
		Extended	3, 3, 4, 5	contimbre
	Cello	Traditional	2, 3, 4, 5, 6	VSL
		Extended	2, 2, 2, 2, 4, 4	contimbre
	Double bass	Traditional	2, 3	MUMS
		Extended	2, 2, 2, 3	contimbre
	Treble viol	Traditional	4, 5	MUMS
	Tenor viol	Traditional	3, 4, 5	MUMS
	Bass viol	Traditional	2, 3, 4	MUMS
	Harp (bowed)	Extended	NA	contimbre
Percussion	Cymbal	Extended	NA	VSL
	Cymbal 2	Extended	NA	VSL
	Cymbal 3	Traditional	NA	VSL
	Snare drum (brush)	Traditional	NA	VSL
	Ratchet	Traditional	NA	VSL
	Thundersheet	Traditional	NA	VSL
	Lion roar	Traditional	NA	VSL
	Feder	Traditional	NA	VSL

For brass, woodwind, and string instruments, extended techniques were generally selected as examples of sounds with various levels of noisy features. Note that, although several of the percussion sounds are produced with traditional techniques, our percussion samples were generally also chosen as examples of sounds with various levels of noisy features. It should also be noted that because the percussion sounds were not included in the linear mixed effects models (see 3.2 in the manuscript), the distinctions made here between "traditional" and "extended technique" for percussion, which are admittedly arguable, do not affect the analyses reported in the paper.
